# Growth performance, nutrient digestibility, fecal microbial diversity and volatile fatty acid, and blood biochemical indices of suckling donkeys fed diets supplemented with multienzymes

**DOI:** 10.1186/s12917-024-03907-1

**Published:** 2024-02-21

**Authors:** Chao Li, Xuan Yue Li, Xiao Bin Li, Chen Ma, Hui Chen, Fan Yang

**Affiliations:** 1 College of Animal Science, Xinjiang Key Laboratory of Herbivore Nutrition for Meat & Milk Production, Xinjiang Agricultural University, Urumqi, 830052 Xinjiang China; 2 Animal Nutrition and Feed Science, College of Animal Science, Xinjiang Agricultural University, Urumqi, Xinjiang 830052 China; 3 Growth and Metabolism of Herbivores, College of Animal Science, Xinjiang Agricultural University, Urumqi, Xinjiang 830052 China

**Keywords:** Multienzymes supplementations, Suckling donkeys, Growth performance, Apparent nutrient digestibility, Microbial diversity

## Abstract

**Background:**

As the foal grows, the amount of breast milk produced by the donkey decreases. In such cases, early supplemental feeding is particularly important to meet the growth needs of the foal. Foals have an incompletely developed gastrointestinal tract with a homogenous microbiota and produce insufficient amounts of digestive enzymes, which limit their ability to digest and utilize forage. Improving the utilization of early supplemental feeds, promoting gastrointestinal tract development, and enriching microbial diversity are the hotspots of rapid growth research in dairy foals. Plant-based feeds usually contain non-starch polysaccharides (NSPs), including cellulose, xylan, mannan, and glucan, which hinder nutrient digestion and absorption. In addition, proteins and starch (both biomolecules) form a composite system mainly through non-covalent interactions. The proteins wrap around the surface of starch granules and act as a physical obstacle, thereby inhibiting water absorption and expansion of starch and decreasing the enzyme's catalytic effect on starch. Glyanase, β-mannanase, β-glucanase, cellulase, protease, and amylase added to cereal diets can alleviate the adverse effects of NSPs. The current study determined the effects of adding multienzymes (glyanase, β-mannanase, β-glucanase, cellulase, protease, and amylase) to the diet of 2-month-old suckling donkeys on their growth performance, apparent nutrient digestibility, fecal volatile fatty acid (VFA) and pH, fecal bacterial composition, and blood biochemical indices.

**Results:**

On day 120 of the trial, fecal samples were collected from the rectum of donkeys for determining bacterial diversity, VFA content, and pH. Moreover, fresh fecal samples were collected from each donkey on days 110 and 115 to determine apparent digestibility. The multienzymes supplementations did not affect growth performance and apparent nutrient digestibility in the donkeys; however, they tended to increase total height gain (*P* = 0.0544). At the end of the study, the multienzymes supplementations increased (*P* < 0.05) the Observed species, ACE, Chao1, and Shannon indices by 10.56%, 10.47%, 10.49%, and 5.01%, respectively. The multienzymes supplementations also increased (*P* < 0.05) the abundance of *Firmicutes*, *Oscillospiraceae*, *Lachnospiraceae*, *Christensenellaceae*, *Christensenellaceae_R-7_group*, and *Streptococcus* in feces, whereas decreased (*P* = 0.0086) the abundance of Proteobacteria.

**Conclusions:**

Multienzymes supplementations added to a basal diet for suckling donkeys can increase fecal microbial diversity and abundance.

## Background

Suckling during lactation is crucial for donkeys because the foal receives vital nutrients from breast milk. With the growth of the foal and the decrease in the amount of breast milk, early supplementary feeding is particularly crucial for meeting the growth needs of the foal. At the same time, in foals, the gastrointestinal tract is not completely developed, the intestinal microbiota is homogenous, the secretion of digestive enzymes is insufficient, and the digestion and utilization of forage is limited. Improving the utilization rate of early supplementary feed, promoting gastrointestinal tract development, and enriching the microbial diversity have become hot topics in the study of rapid growth of suckling foals. Enzyme preparation is a protein composed of amino acids. Supplementing the livestock and poultry diet with this preparation can enhance animal health and improve endogenous enzyme activity and antioxidant, bacteriostatic, and bactericidal functions [1]. Dietary supplementation of a compound enzyme preparation containing amylase, protease, and xylanase can promote the growth of young animals by improving nutrient digestibility and regulating their intestinal flora [2]. Studies have shown that adding a compound enzyme preparation to the feed can supplement the lack of endogenous enzymes, effectively reduce the level of antinutritional factors in feed, improve the feed nutritional value, and promote the healthy growth of animals [3–5].

Plant-based feeds such as corn, oats, and alfalfa usually contain non-starch polysaccharides (NSPs), including xylan, mannan, and glucan. When dissolved in water, NSPs produce sticky substances that hinder the diffusion of substrates and enzymes in the gut, thus preventing nutrient digestion and absorption. At the same time, the high viscosity of feed increases the amount of water consumed by the animal, and a rise in the water content of feces increases nutrient excretion, thereby implying an increase in nutrient consumption. Additionally, the main component of the feed cell wall is cellulose, which is not easily degradable and has a very low utilization rate. Matrices, such as hemicellulose and pectin, wrap around the cellulose skeleton and enclose feed nutrients inside the cell wall. Digestive enzymes that decompose NSPs cannot be secreted in the digestive tract of monogastric animals, and this hinders nutrient absorption. Moreover, protein and starch, both biomolecules, mainly form a composite system through non-covalent interactions. The protein wraps itself on the surface of starch granules, thereby acting as a physical obstruction that inhibits water absorption and expansion of starch and reduces the catalytic effect of enzymes on starch [6–9]. Glyanase, β-mannanase, β-glucanase, cellulase, protease, and amylase added to cereal diets can alleviate the adverse effects of NSPs such as hindering the digestion and absorption of nutrients and increasing the water consumption of the animal. Thus, feeding suckling donkeys on diets supplemented with enzyme products containing glyanase, β-mannanase, β-glucanase, cellulase, protease, and amylase diets is hypothesized to improve their growth performance, gut health, and nutrient digestibility. Moreover, the magnitude of improvement in the aforementioned parameters of suckling donkeys varies depending on the sources of enzymes in the enzyme products. The current study determined the effects of adding multienzymes (glyanase, β-mannanase, β-glucanase, cellulase, protease, and amylase) to the diet of 2-month-old suckling donkeys on their growth performance, apparent nutrient digestibility, fecal volatile fatty acid (VFA) and pH, fecal bacterial composition, and blood biochemical indices.

## Materials and methods

### Animals

Fourteen 2-month-old suckling donkeys (female donkeys, initial body weight = 46.62 ± 2.23 (SE) kg) were obtained from Xinjiang Jinhuyang Animal Husbandry Company Limited. On day 1, the donkeys were weighed and randomly assigned using a randomized complete block design to either of the control group (C) and multienzymes group (T). Each donkey was kept in one stall with a mare, and the floor of the enclosure was made of red brick. The space allowed for two donkeys was 12 m^2^. A single 300-m^2^ activity area was allotted to all donkeys. The average temperatures inside and outside the enclosures were 26℃ and 30℃, respectively, throughout the 4-month study period (from May 1 to August 31, 2021). Body weight and body size (body height, body length, and chest and shank circumference) measurements were taken on a monthly basis. Nutrient digestibility was monitored at 110–115 days of the experiment.

Multienzyme supplementations preparations and supplementation dose.

The compound enzyme preparation was purchased from Yidori Biotechnology Company Limited (Guangdong, China). The preparation included 15,000 U/g xylanase, 400 U/g β-mannanase, 10,000 U/g β-glucanase, 2000 U/g cellulase, 1000 U/g protease, and 300,000 U/g amylase.

### Treatments and experimental design

The study included two diet groups: a control group (C) and a multienzymes group (T). The multienzymes supplementations dosage was 6.5 mg/kg body weight·day^−1^ [10, 11]. The basal diet consisted of the concentrate supplement, alfalfa hay, and corn stover. The basal diet was fed as the control diet, and the test diet was the basal diet containing the multienzymes supplementations. The feed amount of the concentrate supplement was 0.65 kg/100 kg body weight^−1^, and the feed amount was adjusted every 30 days according to the change in the foal’s weight. Alfalfa hay and corn stover were mixed 1:1, crushed (2–3 cm in length), and fed freely. The diet composition (Table 1), nutrition level (Table 2), and feeding management of all female donkeys were identical.

**Table 1:** Composition of concentrate supplement(%)

The raw material	Proportion
Corn	60.00
Soybean meal	30.00
Wheat bran	5.00
CaHPO_4_	2.00
NaCl	0.50
Stone powder	1.00
Premix^a^	1.00
Lysine	0.25
Methionine	0.25

^a^The premix provided the following per kilogram of concentrate supplement: Vitamin A 4200 IU, vitamin B_1_ 0.4 mg, vitamin B_2_ 2 mg, vitamin B_6_ 1.2 mg, vitamin C 20 mg, vitamin D_3_ 880 IU, vitamin E 500 IU, Pantothenic acid 10 mg, Nicotinamide 100 mg, Copper 25 mg, Iron 107 mg, Manganese 81 mg, Zinc 74 mg, Iodine 6 mg, Selenium 14 mg, Cobalt 3 mg, Choline chloride 120 mg

**Table 2:** Nutrient levels of corn stalk, alfalfa hay, and supplements (dry matter, DM basis)

Nutrient	Corn stalk	Alfalfa hay	Supplement
Dry Matter, DM (%)	95.08	94.27	93.51
Organic Matter, OM (%)	97.49	95.47	94.21
Gross Energy, GE (MJ/kg)	16.68	17.37	18.05
Crude Protein, CP (%)	5.26	12.25	17.83
Neutral Detergent Fiber, NDF (%)	77.13	60.84	24.51
Acid Detergent Fiber, ADF (%)	42.60	54.12	5.53
Calcium, Ca (%)	0.36	1.50	1.16
Phosphorus, P (%)	0.16	0.25	1.08

### Sample collections

On day 1 and day 120 of the study, blood samples of all donkeys were collected through the jugular vein. The plasma was prepared and stored at -20 °C for determining the blood biochemical indices.

On day 120 before the morning feeding, the donkey foals were cleaned around the anus and fecal samples were collected through the rectum by using disposable gloves and a cotton swab. The collected samples were snap-frozen in liquid nitrogen and stored at -80 °C for determining the microbial composition and VFA concentration.

Nutrient digestion tests were conducted and feed and fecal samples were collected on days 111 through 115. During the nutrient digestion period, each donkey foal’s feed was weighed daily before morning feeding (09:00). The leftovers of forage in the trough of each donkey foal were collected before morning feeding (09:00) the next day, and the amount of leftovers was weighed, the amount of food consumed by each donkey foal was calculated every day. 500 g of forage and 500 g of concentrate supplement fed during the digestion tests were collected according to the 5-point sampling method, the forage samples and concentrate supplement fed were dried naturally, weighed, pulverized, encapsulated in a sealed bag, and kept sealed for testing.

### Chemical analysis

Fecal and forage samples were dried in an oven at 65°C for 48 h, ground, and passed through a 0.4-mm sieve by using a centrifugal mill before chemical analysis. All samples were analyzed for dry matter (DM), gross energy (GE), crude protein (CP), organic matter (OM), calcium (Ca), and phosphorus (P) contents and neutral detergent fiber (NDF) and acid detergent fiber (ADF) levels.

The collected concentrate, forage, forage residue, and dried fecal samples were pulverized in a grinder (multifunctional high-speed grinder, 400 *g* upright type; Tohe Electromechanical Technology (Shanghai) Co., Ltd., Shanghai, China), passed through a 0.4-mm sieve, and analyzed using the Chinese national standard routine nutrient content determination [12] method to determine DM, crude ash (ash), CP, Ca, P, and OM contents. These values were calculated as OM = (100% − ash%). The NDF and ADF levels were determined according to the method of Van Soest by using an automated fiber analyzer (ANKOM-2000, USA; Shanghai Longjie Instrument Equipment Co., Ltd., Shanghai, China) [13]. The GE was determined using a highly accurate and precise calorimeter type OR2014 (Shanghai Ou Rui Instrument) Equipment Co., Ltd., Shanghai, China).

### Microbial DNA extraction from fecal samples

Total genomic DNA from the samples was extracted using the CTAB/SDS method [14]. DNA concentration and purity were assessed on 1% agarose gels. DNA was diluted to 1 ng/µL by using sterile water. The DNA was submitted to Novogene (Beijing, China) for processing.

### V3-V4 16S rRNA gene amplification and sequencing

The universal primers 341F 5′-CCTAYGGGRBGCASCAG-3′ and 806R 5′-GGACTACNNGGGTATCTAAT-3′ were used to obtain PCR amplicons for paired-end sequencing on an Illumina MiSeq platform at Novogene (China).

16S rRNA genes were amplified using the specific primer with the barcode. All PCR reactions were carried out in a 30-µL reaction mixture containing 15 µL of Phusion® High-Fidelity PCR Master Mix (New England Biolabs), 0.2 µM of forward and reverse primers, and approximately 10 ng template DNA. Thermal cycling consisted of initial denaturation at 98℃ for 1 min, followed by 30 cycles of denaturation at 98℃ for 10 s, annealing at 50℃ for 30 s, elongation at 72℃ for 30 s, and extension at 72℃ for 5 min [14–16].

Using the Illumina TruSeq DNA PCR-Free Library Preparation Kit (Illumina, USA), sequencing libraries were generated following the manufacturer’s recommendations, and index codes were added. The library quality was evaluated using the Qubit@ 2.0 Fluorometer (Thermo Scientific) and Agilent Bioanalyzer 2100 system. Then, the library was sequenced on an Illumina NovaSeq platform, and 250-bp paired-end reads were generated.

### Bioinformatics analyses

The obtained paired-end reads were assigned to samples based on their unique barcodes, truncated by cutting off the barcodes and primer sequences, and merged using FLASH (Version 1.2.7)^1^ [14]. The raw tags were quality-filtered under specific filtering conditions to obtain high-quality clean tags [17] according to the QIIME (Version 1.9.1)^2^ [15] quality control process. Next, the tags were compared with the reference database (Silva database)^3^ by using the UCHIME algorithm^4^ to detect chimera sequences, which were then removed [18, 19]. Then, the effective tags were finally obtained.

The sequences were analyzed using Uparse software (Uparse Version 7.0.1001)^5^ [20]. Sequences with ≥ 97% similarity were assigned to the same operational taxonomic units (OTUs). Representative sequence for each OTU was screened for further annotation [21, 22].

Alpha diversity was applied for analyzing the complexity of species diversity for a sample on the basis of six indices, namely Observed species, Chao1, Shannon, Simpson, ACE, and Good’s coverage. All these indices were calculated using QIIME (Version 1.9.1) and displayed using R software (Version 2.15.3).

Beta diversity analysis was performed to evaluate differences in species complexity in the samples. Beta diversities on both weighted and unweighted Unifrac were calculated using QIIME (Version 1.9.1). Cluster analysis was preceded by principal component analysis (PCA), which was used to reduce the dimension of original variables by using the FactoMineR package and ggplot2 package in R software (Version 2.15.3). Principal coordinate analysis (PCoA) was performed to obtain principal coordinates and visualize complex, multidimensional data.

Linear discriminant analysis (LDA) effect size (LEfSe) analysis was performed using LEfSe software with a default screening value of 4 for the LDA score. Using R software, metastats analysis was performed at each classification level (phylum, class, order, family, genus, and species) by conducting the permutation test between groups to obtain *P* values. Then, the Benjamini and Hochberg false discovery rate method was used to correct for these *P* values to obtain q values [22].

### Determination of VFA in fecal samples

The concentrations of VFA (e.g., acetate, propionate, butyrate, isobutyrate, valerate, and isovalerate) in feces were determined through gas chromatography. Fecal subsamples were stored at − 80 ℃, thawed, and homogenized. A fecal subsample (10 g) was mixed with 10 mL of ultrapure water, vortexed at 4 ℃ for 30 min, and filtered through four layers of gauze. The entire filtrate was centrifuged at 5000 × *g* for 10 min, and the supernatant was placed at 4 ℃ for 12 h. Next, 0.6 mL of the supernatant was mixed with 0.6 mL of 10% trichloroacetic acid (Sigma Aldrich (Shanghai) Trading Co.) and 0.1 mL of 60 mmol of internal standard solution (4-methylpentanoic acid, Sigma Aldrich (Shanghai) Trading Co.). The mixture was vortexed, allowed to stand for 20 min at 4 ℃, and centrifuged at 20,000 × *g* for 15 min. Finally, 1 µL of this solution was used for analysis. The gas chromatograph (Agilent 7890A, Agilent, Agilent Technologies (China) Ltd, Beijing, China) was equipped with an HP-FFAP (50 m × 0.20 mm (internal diameter) × 0.33 µm (film thickness)) capillary column (Agilent Technologies (China) Ltd, China). The following parameters were established: chromatograph oven temperature was increased by 10 ℃/min from 60 ℃ to 220 ℃ for 12 min, and the injector and detector were maintained at 240 ℃ and 280 ℃, respectively. Nitrogen was used as carrier gas at a flow rate of 5.0 mL/min.

### Determination of blood biochemical indices

At the end of the study, blood samples were collected. In total, 13 blood biochemical indices were detected: total protein (TP), albumin (ALB), urea (UREA), creatinine (CREA), glucose (Glu), total bile acid (TBA), triglycerides (TG), alanine aminotransferase (ALT), alkaline phosphatase (ALP), γ-glutamyl transferase (γ-GT), cholinesterase (CHE), creatine kinase (CK), and lactate dehydrogenase (LDH). The automatic biochemical analyzer (model BS-240VET) (China Guangdong Shenzhen Mindray Bio-Medical Electronics Company Limited) was used for detecting the blood biochemical indices by using the method described on the kit. The detection indices and methods are presented in Table 3.

**Table 3:** Detection indices and methods

Serial number	Indices	Detection methods
1	Total protein (TP) g/L	Biuret method
2	Albumin (ALB) g/L	Bromocresol green method
3	Urea (UREA) mmol/L	UV-glutamate dehydrogenase method
4	Creatinine (CREA) μmol/L	The sarcosine oxidase method
5	Glucose (Glu) mmol/L	Glucose oxidase method
6	Total bile acid (TBA) μmol/L	Vanadate oxidation method
7	Triglycerides (TG) mmol/L	Oxidase method
8	Alanine aminotransferase (ALT) U/L	IFCC method
9	Alkaline phosphatase (ALP) U/L	AMP buffer method
10	γ-Glutamyl transferase (γ-GT) U/L	IFCC method
11	Cholinesterase (CHE) U/L	Butyryl thiocholine method
12	Creatine kinase (CK) U/L	IFCC method
13	Lactate dehydrogenase (LDH) U/L	IFCC method

Globulin is the calculated value

### Statistical analyses

Data on growth performance, apparent nutrient digestibility, alpha diversity, VFA and pH of fecal samples and blood biochemical indices of donkey foals were statistically analyzed using the SAS 9.4 general linear model procedure with the model: Yij = µ + τi + εij, where Yij is the response variable, µ is the overall mean, τi is the experimental diet (i = control group or multienzymes group), and εij is the residual error. In this model, diet was considered as a fixed effect, and the animal was considered as a random effect. The Shapiro–Wilk test was used to normalize all data. All data were analyzed using the *t*-test. The least-squares means were reported, and *P* < 0.05 was declared significant.

## Results

### Growth performance of experimental diet-fed donkey

Table 4 presents the effects of multienzymes supplementations on body weight and size. The body weight and size of the donkeys at the end of day 120 were not significant.

**Table 4:** Growth performance of experimental diet-fed donkeys

Items		Control group	Multienzymes group	SEM	*P* value
Body weight (kg)	0 days	46.33	46.90	1.50	0.8622
	120 days	101.67	105.60	2.58	0.4779
	Total weight gain	55.33	58.70	1.66	0.3380
Body height (cm)	0 days	90.53	87.66	1.10	0.2079
	120 days	107.87	108.86	0.83	0.5521
	Total height gain	17.33	21.20	1.01	0.0544
Body length (cm)	0 days	73.05	72.88	1.73	0.9639
	120 days	97.18	98.24	0.83	0.5566
	Total length gain	24.13	25.36	1.97	0.7739
Chest (cm)	0 days	81.00	79.62	1.43	0.6554
	120 days	102.88	103.18	1.13	0.9035
	Total chest gain	21.88	23.56	1.33	0.6031
Shank circumference (cm)	0 days	11.25	11.22	0.17	0.9364
	120 days	13.47	13.90	0.19	0.2803
	Total shank circumference gain	2.22	2.68	0.19	0.2557

### Apparent nutrient digestibility in experimental diet-fed donkeys

Table 5 lists the effects of multienzymes supplementations on apparent nutrient digestibility in donkeys. Multienzymes supplementations had no significant effect on apparent nutrient digestibility in the donkeys.

**Table 5:** Apparent nutrient digestibility in experimental diet-fed donkeys

Items	Control group	Multienzymes group	SEM	*P* value
Dry matter, DM (%)	56.66	58.91	1.40	0.4622
Organic matter, OM (%)	63.60	63.99	1.31	0.8820
Gross energy, GE (%)	73.51	76.94	1.31	0.1848
Crude protein, CP (%)	60.40	61.83	1.24	0.6208
Neutral detergent fiber, NDF (%)	35.59	39.66	2.02	0.3491
Acid detergent fiber, ADF (%)	34.00	36.59	2.23	0.5998
Calcium, Ca (%)	56.51	59.28	3.03	0.6790
Phosphorus, P (%)	44.50	43.66	5.56	0.9462

### Alpha diversity

Table 6 presents the effects of multienzymes supplementations on the alpha diversity of fecal bacteria in the donkeys. The multienzymes supplementations basal diet increased Observed species (*P* = 0.010), Shannon (*P* = 0.046), Chao1 (*P* = 0.010), and ACE (*P* = 0.011).

**Table 6:** Alpha diversity of fecal bacteria in experimental diet-fed donkeys

Items	Control group	Multienzymes group	SEM	*P* value
Observed species	1610.2	1780.2	34.79	0.010
Shannon	8.18	8.59	0.11	0.046
Simpson	0.99	0.99	0.01	0.212
Chao1	1621.95	1792.13	34.90	0.010
ACE	1645.64	1817.87	35.40	0.011
PD whole tree	151.03	141.9	6.64	0.524

### Veen figure

**Fig. 1:**
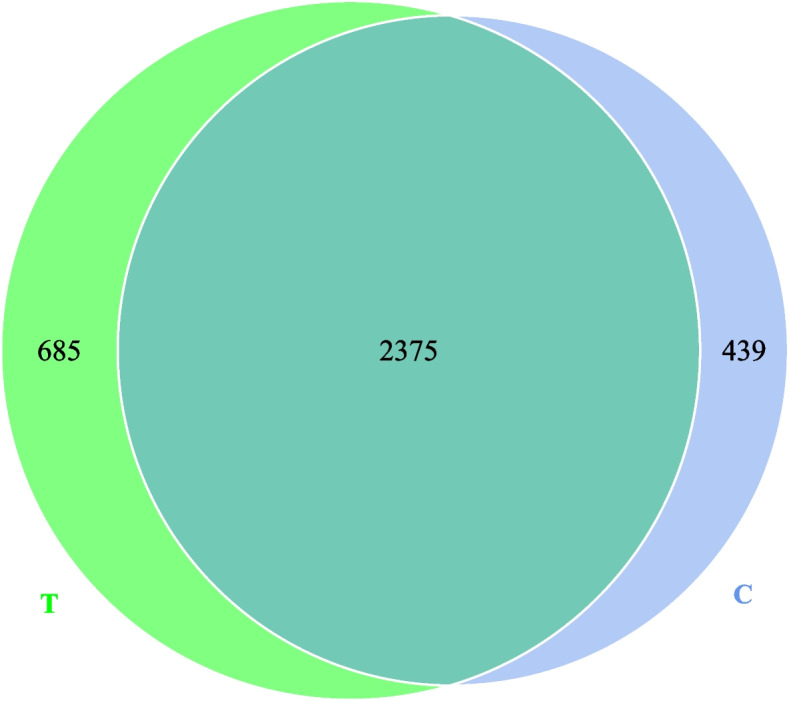
Veen figure of fecal bacteria in experimental diet-fed donkeys. T represents the multienzymes group, and C represents the control group

Figure 1 illustrates the effects of multienzymes supplementations on the Veen figure of fecal bacteria in the donkeys. In total, 2375 species were observed, with 439 and 685 species in the control and experimental groups, respectively.

### Principal component analysis and principal coordinate analysis

**Fig. 2:**
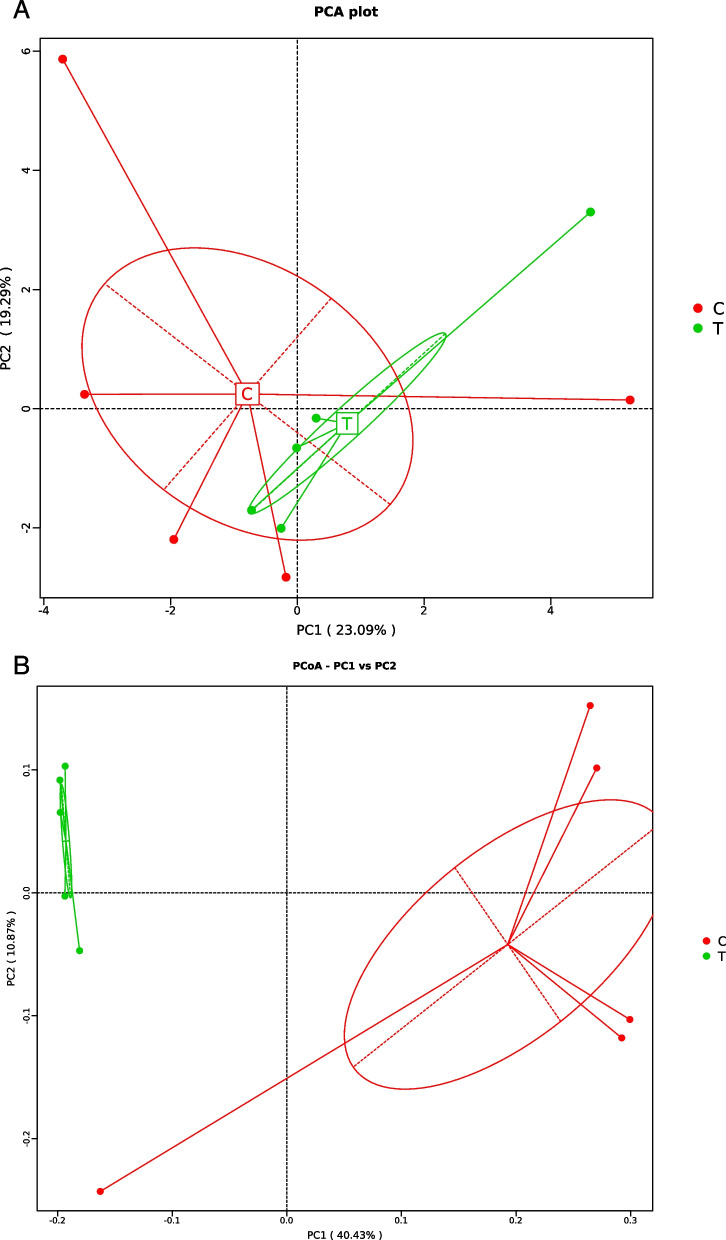
Effects of multienzymes supplementations on fecal bacteria PCA (**A**) and PCoA (**B**) in donkeys. **A** Red C represents the control group, and green T represents the multienzymes group. **B** red C represents the control group, and green T represents the multienzymes group

The PCA is used to reduce the dimensionality of multidimensional data based on the relative abundance distribution of amplicon sequence variant (ASV) so as to extract the most important elements and structures in the data. Figure 2A presents the PCA results of the effects of multienzymes supplementations on fecal bacteria in donkeys. The PCA could extract two coordinate axes that reflect the difference between samples to the greatest extent. This ensured that the difference in multidimensional data can be reflected on the two-dimensional coordinate graph, and then, the simple laws under the background of complex data can be revealed. The more similar the community composition of the samples, the closer they are in the PCA plot.

The PCoA is applied to extract the most crucial elements and structures from multidimensional data through a series of eigenvalues and eigenvector sorting. Figure 2B presents the PCoA results of the effects of multienzymes supplementations on fecal bacteria in donkeys. We conducted the PCoA based on the weighted and unweighted Unifrac distances. The principal coordinate combination with the largest contribution rate was selected for display. The closer the sample distance is, the more similar the species composition is. Therefore, samples with a high community structure similarity tend to be clustered together and those with great community differences tend to stay farther apart.

### Phylum, family, genus, and species level abundance

**Fig. 3:**
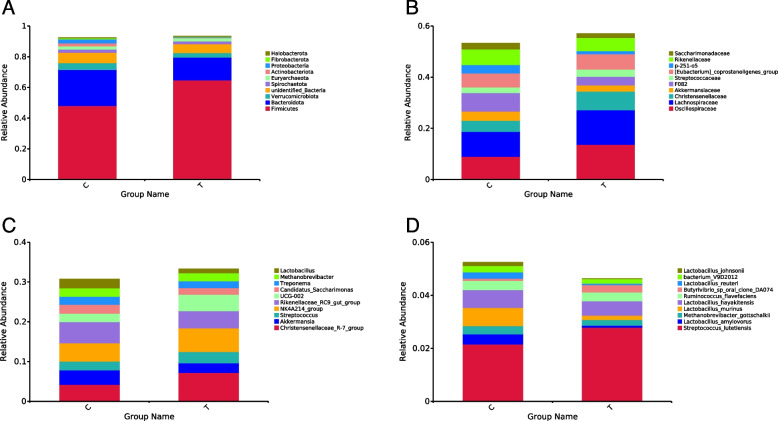
Effects of multienzymes supplementations on the relative abundance of fecal bacterial in donkeys at phylum (**A**), family (**B**), genus (**C**), and species (**D**) levels. **A** indicates the relative abundance histogram of the top 10 bacterial phyla in donkey foal feces. **B** indicates the relative abundance histogram of the top 10 bacterial families in donkey foal feces. **C** indicates the relative abundance histogram of the top 10 bacterial genera in donkey foal feces. **D** indicates the relative abundance histogram of the top 10 bacterial species in donkey foal feces

Figure 3A presents the effects of multienzyme supplementations on the relative abundance of fecal bacterial phyla in donkeys. The multienzymes supplementations basal diet increased the relative abundance of Firmicutes (*P* < 0.01). On the other hand, the multienzymes supplementations basal diet reduced the relative abundance of Bacteroidota, Verrucomicrobiota, unidentified_Bacteria, Spirochaetota, Euryarchaeota, Actinobacteriota (*P* < 0.05), Proteobacteria (*P* < 0.01), Fibrobacterota, and Halobacterota.

The effects of multienzyme supplementations on the relative abundance of the fecal bacterial family in donkeys are presented in Fig. 3B. The multienzymes supplementations basal diet increased the relative abundance of Oscillospiraceae (*P* < 0.05), Lachnospiraceae (*P* < 0.05), Christensenellaceae (*P* < 0.05), Streptococcaceae, and [Eubacterium]_coprostanoligenes_group. On the other hand, the multienzymes supplementations basal diet reduced the relative abundance of Akkermansiaceae, F082, p-251-o5, Rikenellaceae, and Saccharimonadaceae. None of them were significantly different.

Figure 3C presents the effects of multienzyme supplementations on the relative abundance of the fecal bacterial genera in donkeys. The multienzymes supplementations basal diet increased the relative abundance of *Christensenellaceae_R-7_group* and *Streptococcus* (*P* < 0.05). On the other hand, the multienzymes supplementations basal diet reduced the relative abundance of *Akkermansia*, *NK4A214_group*, *Rikenellaceae_RC9_gut_group*, *UCG-002* (*P* < 0.05), *Candidatus_Saccharimonas*, *Treponema*, *Methanobrevibacter*, and *Lactobacillus* (*P* < 0.01).

Figure 3D presents the effects of multienzyme supplementations on the relative abundance of fecal bacterial species in donkeys. The multienzymes supplementations basal diet increased the relative abundance of *Streptococcus_lutetiensis*, *Lactobacillus_amylovorus*, *Methanobrevibacter_gottschanlkii*, *Lactobacillus_murinus*, *Lactobacillus_hayakitensis*, *Ruminococcus_flavefaciens*, *Butyrivibrio_sp_oral_clone_DA074*, *Lactobacillus_reuteri*, *bacterium_V9D2012*, and *Lactobacillus_johnsonii*, but the result was not significant.

### LEfSe analysis

**Fig. 4:**
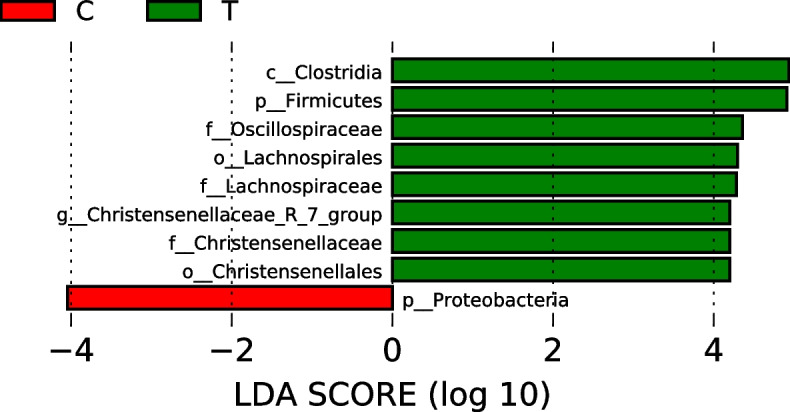
LEfSe is an analytical tool used for discovering and interpreting high-dimensional biomarkers (genes, pathways, and taxa). It can be used to compare two or more groups. This tool clarifies about statistical significance and biological correlation and can identify statistically different biomarkers between groups. Green represents species with significant abundance difference in the multienzymes group, and red represents species with significant abundance difference in the control group

The LEfSe results of effects of multienzymes supplementations on fecal bacteria in donkeys are presented in Fig. 4. In the experimental group, eight bacteria were found at each classification level: Clostridia, Firmicutes, Oscillospiraceae, Lachnospirales, Lachnospiraceae, *Christensenellanceae_R_7_group*, Christensenellaceae, and Christensenellales. In the control group, only one species, Proteobacteria, was identified.

### The Tax4Fun

**Fig. 5:**
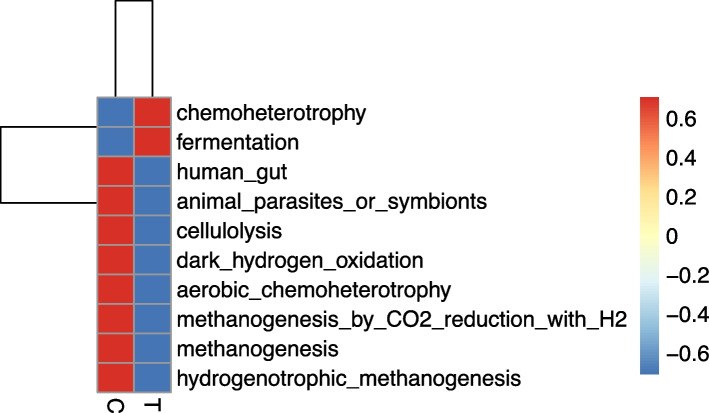
Tax4Fun is an R program package based on the 16S Silva database for the functional prediction of the microbial community composition of intestinal, soil, and other environmental samples, with high prediction accuracy. Functions were predicted with Tax4Fun by using the nearest neighbor method based on minimum 16S rRNA sequence similarity. In this method, the 16S rRNA gene sequence of the prokaryote whole genome is extracted from the KEGG database and compared with the SILVA SSU Ref NR database by using the BLASTN algorithm (BLAST bitscore > 1500). Then, the correlation matrix is established, and the prokaryotic whole genome functional information annotated by UProC and PAUDA in the KEGG database is compared with the SILVA database to realize the functional annotation of the SILVA database. Sequencing samples were clustered with the SILVA database sequence as the reference sequence to obtain OTU, and then, the functional annotation information was obtained

In our study, Tax4Fun was used to precisely predict the functional properties of the fecal samples. Figure 5 displays a functional annotation clustering map of donkey fecal samples generated using Tax4Fun. Functional information of 10 bacterial species in the foal fecal samples was identified. The multienzymes group exhibited a significant increase in two species, which were associated with chemotaxis and fermentation functions.

### VFA and pH of fecal samples from experimental diet-fed donkeys

Table 7 presents the effects of multienzymes supplementations on the VFA and pH of feces in donkeys. The multienzymes supplementations basal diet had no significant effect on acetic acid, propionic acid, butyric acid, valeric acid, isovaleric acid, total VFA, and pH in foal feces.

**Table 7:** VFA and pH of fecal samples from experimental diet-fed donkeys

Items	Control group	Multienzymes group	SEM	*P* value
Acetate mg/g	2.89	2.53	0.30	0.582
Propionate mg/g	0.78	0.60	0.10	0.382
Isobutyrate mg/g	0.12	0.10	0.01	0.308
Propionate mg/g	0.35	0.23	0.06	0.385
Isovalerate mg/g	0.18	0.15	0.02	0.433
Valerate mg/g	0.09	0.06	0.01	0.137
Total VFA mg/g	4.40	3.67	0.45	0.796
pH	6.80	6.79	0.02	0.892

### Blood biochemical indices

Table 8 presents the effects of multienzymes supplementations on blood biochemical indices in donkeys. The multienzymes supplementations basal diet reduced (*P* < 0.05) the blood biochemical indices of Glu. The multienzymes supplemented basal diet had no significant effect on TP, ALB, globulin (GLB), UREA, CREA, TBA, TG, ALT, ALP, γ-GT, CHE, CK, and LDH.

**Table 8:** Blood biochemical indices in experimental diet-fed donkeys

Items	Control group	Multienzymes group	SEM	*P* value
Total protein (TP) g/L	61.94	54.14	2.27	0.095
Albumin (ALB) g/L	30.66	26.64	1.32	0.137
Globulin (GLB) g/L	31.28	27.50	1.32	0.198
Urea (UREA) mmol/L	8.39	7.94	0.35	0.557
Creatinine (CREA) μmol/L	47.58	42.36	2.80	0.381
Glucose (Glu) mmol/L	5.76	4.64	0.27	0.030
Total bile acid (TBA) μmol/L	26.66	21.96	1.59	0.148
Triglycerides (TG) mmol/L	0.40	0.37	0.03	0.541
Alanine aminotransferase (ALT) U/L	5.80	5.52	0.50	0.796
Alkaline phosphatase (ALP) U/L	303.46	260.40	22.02	0.358
γ-Glutamyl transferase (γ-GT) U/L	43.04	50.82	4.06	0.368
Cholinesterase (CHE) U/L	8089.20	8013.40	265.11	0.897
Creatine kinase (CK) U/L	371.78	411.00	38.98	0.646
Lactate dehydrogenase (LDH) U/L	414.20	440.64	38.45	0.753

## Discussion

Supplementing a diet containing multienzymes can reduce the antinutritional effect of NSPs and protein inhibitors in feed and improve nutrient digestibility, thereby improving the growth performance and feed utilization efficiency of young animals [23]. Owusu et al. found that 0.1% and 0.2% complex enzymes added to a broiler diet significantly improved the growth performance of 42-day-old broilers and reduced the feed consumption rate [24]. Han et al. added an acid protease-containing complex enzyme preparation to the diet of nursing pigs. This multienzyme preparation significantly improved the activities of related digestive enzymes in the intestines of these pigs and their growth performance [25]. Du et al. found that compared with single enzyme preparations, compound enzyme preparations had a better effect on the growth performance of weaned piglets and the utilization rate of nitrogen and phosphorus [26]. Ai et al. reported that NSP enzymes can inhibit the secretion of growth- and metabolism-related hormones. NSP enzymes added to the diet can improve the digestion and metabolism of animals, the metabolic hormone level in peripheral blood in vitro, and the feed utilization rate and growth rate [27]. Murray et al. showed that 6222 IU/g cellulase, 1039 IU/g xylanase, and 2156 IU/g β-glucanase promoted body weight gain in Welsh-cross pony geldings [8]. In this study, the complex enzyme preparation, mainly composed of xylanase, β-mannanase, β-glucanase, cellulase, protease, and amylase, was added to the foal diet, thereby promoting the digestion of NPS cellulose, protein, and starch in the diet [2]. The concentrate supplement of donkeys was mainly composed of corn and soybean meal. The multienzymes group had higher body weight and size than the control group, but the result was not significant. Enzymes such as NSP enzymes release more nutrients from the cell wall of cereals, and protease decomposes the soybean antigen protein. This protein cannot be easily decomposed into small peptides and amino acids by mammals. Once decomposed, the soybean antigen protein is easily absorbed, which increases the nutrient concentration, and promotes gastrointestinal tract development and digestive enzyme activity, there by improving the growth and development of donkey foals.

In our study, the digestibility of DM, CP, NDF, and ADF was higher in the multienzymes group than in the control group, but the result was not significant. Hainze et al. found that the addition of cellulase to equine rations improved the digestibility of NDF and ADF [7]. Salem et al. found that the addition of cellulase and xylanase to bran and oat straw rations improved the digestibility of DM, OM, CP, NDF, and ADF in Quarter Horse [9]. Enzymes such as cellulase and glucanase are crucial for degrading indigestible cellulose and antinutritional factors in the diet, and thus, they can improve the digestibility and absorption rate of dietary nutrients. These results revealed that exogenous cellulase added to diets could improve the feed utilization rate by improving cellulose digestion and absorption in experimental animals. Cellulase can directly destroy the cellulose structure after reaching the gastrointestinal tract and improve the feed utilization rate for cultured animals. Our study suggests that enzyme complexes may benefit nutrient digestibility and performance by first decomposing the plant cell wall structure and then releasing the cell wall nutrients for animal use. The increase in nutrient digestibility is consistent with the increase in the body weight and size of the donkeys.

Complex dynamic microbial communities present in the large intestine (cecum and colon) of monogastric herbivores have a vital role in nutrient digestion, absorption, and metabolism as well as in physiological and immune aspects. Early colonization of intestinal microbes is fundamental for animal growth and development and full play of later production performance. Increasing attention has been concentrated on the effects of enzymes on intestinal microorganisms and bacteria. Su and Yao reported that enzymes affect intestinal microorganisms through three pathways [28]. Lysozymes, lysozyme-like glycoside hydrolases, and other glycoside hydrolases can kill harmful bacteria in the gastrointestinal tract of animals. For example, xylanase degrades xylan and produces xylo-oligosaccharide, which is used by co-microorganisms to promote microbial growth [28]. Enzymes can affect the gut microbiota by interfering with microbial networking. In our study, feeding multienzymes supplementations significantly changed the bacterial diversity in donkey feces and significantly increased ACE, Chao1, and Shannon indices. This is consistent with the research results of Yang et al. in broilers [29]. With the addition of exogenous enzymes to feed, the bacterial diversity index improved in feces. However, Zhao et al. demonstrated that the complex enzyme preparation had no significant effect on the bacterial diversity of Yili goose cecum [30]. This difference may be related to the different types of enzyme preparations and the different feeding objects.

We statistically analyzed the top 10 strains at each classification level in donkey feces. Dietary supplementation of multienzymes could significantly or extremely significantly increase the abundance of Firmicutes, Oscillospiraceae, Lachnospiraceae, Christensenellaceae, *Christensenellaceae_R-7_group*, and *Streptococcus*, whereas it significantly decreased the abundance of Proteobacteria. Park et al. demonstrated that under the tested conditions, no significant differences were observed in the fecal bacterial composition of weaned pigs after the addition of multienzymes supplementations [31]. In this study, we found that Oscillospiraceae, Lachnospiraceae, Christensenellaceae, *Christensenellaceae_R-7_group*, and *Streptococcus*, whose abundance had significantly increased, all belonged to Firmicutes. The results indicated that multienzymes supplementations can improve the structure and abundance of intestinal bacteria, promote beneficial bacteria, and inhibit harmful bacteria (Proteobacteria, C = 2. 61% and T = 0.38%, *P* = 0. 0086) [32]. Our results exhibited the same result that although multienzymes supplementations could significantly change the bacterial abundance in feces, they have the same effect on the customized flora in the intestinal mucosa of donkeys. Therefore, whether we need to conduct further research to explore the reasons for the change in the coliform abundance of donkey colts induced by exogenous enzyme preparation is unclear in this experiment.

In our study, LEfSe results exhibited statistically different nine biomarker species; of them, eight were in the multienzymes group and all these bacteria belonged to Firmicutes. Tax4Fun results revealed that OTU was clustered with the SILVA database sequence, which was used as a reference sequence. Then, the functional annotation information of bacteria was obtained. In our study, among the top 10 bacterial functions, chemoheterotrophy and fermentation exhibited a significant correlation with complex enzyme supplementation. Chemoheterotrophs are a group of microorganisms that use organic compounds as carbon sources, energy sources, and electron donors. They include most known bacteria and archaea, all actinomycetes, fungi, and protozoa. Through respiration or fermentation (an inefficient productivity reaction), microbes capture energy [33]. In fermentation, electrons generated through incomplete oxidation of organic substrates are directly delivered to endogenous incomplete decomposition products without passing through the electron transport system. These are then reduced to fermentation products, thereby generating a small amount of ATP through substrate level phosphorylation [34]. According to the different types of fermentation products generated, ethanol fermentation and lactic acid fermentation are two basic forms of fermentation. In our study, exogenous enzyme addition promoted the potential chemoheterotrophy and fermentation functions of intestinal bacteria in donkeys. This indicates that the multienzymes supplementations change the structure of the intestinal bacterial community and eventually cause a change in their function. Compared with the control group, the multienzymes supplementations can improve the fermentation function of donkeys and increase the utilization rate of the intestinal fiber feed, which is consistent with the high NDF and ADF digestibility of the multienzymes group.

Microbial fermentation mainly occurs in the cecum and colon of donkeys and is a crucial player in the intestinal health of donkeys. It can produce various VFAs, mainly including formic acid, acetic acid, propionic acid, and butyric acid, thereby effectively inhibiting the reproduction of harmful bacteria and enhancing intestinal nutrient absorption [35]. In Li et al.’s study, acetic acid, propionic acid, and total VFA concentrations in the cecum and colon significantly increased in pigs fed the compound enzyme-supplemented diet than in those fed the diet without enzymes [36]. Long et al. reported that dietary multienzymes supplementations tended to increase acetic acid content in the colon [37]. Yi et al. also reported that dietary multienzymes supplementations could effectively enhance VFA contents and improve the health status of weaned pigs [2]. In our study, the added complex enzymes had no significant effect on VFA and pH. Sorensen et al. suggested that dietary composition is the dominant factor influencing the ratio of the fecal VFA concentration and composition [38]. Exogenous enzymes added to different diets have different effects on VFA production in the animal digestive tract [39]. Mejicanos et al. reported that xylanase added to the coarse white-canola meal of weaned piglets increased the acetic acid concentration from 47.4 to 55.3 mM, whereas that added to a white-regular diet did not increase the concentration [39]. Palframan et al. reported that xylanase addition increased the digestibility of cereal starch in the stomach and reduced butyric acid production in the hindgut, which was responsible for increased intestinal pH [40]. The butyric acid concentration in donkey feces in this study may be low due to the aforementioned reasons. Exogenous enzyme preparations can affect animal feed digestibility to a certain extent. On the basis of metabolites such as VFAs, we can evaluate fiber feed utilization for herbivorous livestock. However, it should also be noted [41] that when using VFA to evaluate the degradation of forage crude fiber, the VFA metabolism rate, the accuracy of determination, and measurements of the presence of enzymes and enzyme-encoding genes, butyrate production would have helped to clarify the impact of xylanase and different diets according to their fiber content on the intestinal microbiota and fermentability pattern.

The difference in blood biochemical indices is closely related to the change in metabolism and nutrition status and disease occurrence. Serum total protein, albumin, and globulin can reflect protein digestion and absorption in animals, maintain the colloidal osmotic pressure of the body, increase the protein content, and improve the immunity of the body accordingly. The body’s nutritional level can be enhanced, and the ability to resist the influence of an external adverse environment can be improved. Blood urea nitrogen and creatinine are crucial renal function markers and can indirectly reflect the protein utilization efficiency in animals to a certain extent. In our study, under the condition of the same dietary composition and nutrient level, CP obtained from the diets of donkeys met the body’s needs for protein metabolism. The added exogenous enzymes had no significant effect on protein-related metabolites in the plasma. Blood glucose content reflects the amount of glucose absorbed into blood by the intestinal tract, which is directly related to the digestibility of dietary carbohydrates. Most studies have shown that enzyme preparations can improve starch digestibility. Starch is absorbed into the blood in the form of glucose after decomposition, thus increasing the blood glucose concentration [42]. The mechanism of intestinal glucose absorption is complex. Absorption is generally inferred to be suitable for the metabolic needs of the body. Blood glucose concentration is regulated by both absorption and metabolism. In our study, in the presence of the same carbohydrate source with the same structure, the blood glucose concentration of donkey colts in the multienzymes group decreased significantly. Whether this is related to the hunger level of donkeys when collecting blood samples on an empty stomach in the morning remains unclear and requires to be determined through in-depth research.

## Conclusions

In conclusion, multienzymes supplementations added to a basal diet for suckling donkeys improved their fecal microbiota diversity. The abundance of *Firmicutes*, *Oscillospiraceae*, *Lachnospiraceae*, *Christensenellaceae*, *Christensenellaceae_R-7_group*, and *Streptococcus* increased in the feces of donkeys. Exogenous enzyme addition promoted the potential chemoheterotrophy and fermentation functions of intestinal bacteria in the donkeys. However, the multienzyme supplementations neither affected the growth performance, nutrient digestibility, VFA, and pH of donkey feces nor their blood biochemical indices.

## Data Availability

All data generated or analyzed during this study are included in this published article. The datasets generated during the current study are available in the  NCBI database [accession number: PRJNA985446. https://www.ncbi.nlm.nih.gov/sra/PRJNA985446].
